# A Case of Neuromyelitis Optica Spectrum Disorder Complicated by Posterior Reversible Encephalopathy Syndrome With Pre-existing Sjögren's Syndrome and Autoimmune Hepatitis

**DOI:** 10.7759/cureus.78098

**Published:** 2025-01-27

**Authors:** Yumiko Fujiwara, Nobuyuki Mori, Mari Fukuda, Shunsuke Tamaki, Akihiro Furuta

**Affiliations:** 1 Department of Radiology, Osaka Red Cross Hospital, Osaka, JPN; 2 Division of Neurology, Kobe University Graduate School of Medicine, Kobe, JPN; 3 Department of Neurology, Osaka Red Cross Hospital, Osaka, JPN

**Keywords:** autoimmune disease, autoimmune hepatitis, neuromyelitis optica spectrum disorder, posterior reversible encephalopathy syndrome, sjögren’s syndrome

## Abstract

Neuromyelitis optica spectrum disorder (NMOSD) is an autoimmune disease of the central nervous system (CNS). The coexistence of NMOSD and other autoimmune diseases is well recognized. Additionally, rare cases of NMOSD complicated by posterior reversible encephalopathy syndrome (PRES) have been reported. We present here a case of a young female who developed NMOSD in the clinical course of Sjögren's syndrome (SS) and autoimmune hepatitis (AIH), and who then rapidly developed PRES. A 28-year-old woman was admitted to our hospital with complaints of nausea and singultus, resulting in difficulty eating. She had been experiencing numbness from her neck to her upper arms prior to admission, and she also developed a decrease in visual acuity in her left eye after admission. Brain magnetic resonance imaging (MRI) revealed optic neuritis and a lesion in the area postrema. Based on these imaging findings and those symptoms described as acute myelitis, she was tentatively diagnosed with NMOSD, and treatment with steroids and plasmapheresis was initiated. The following day, however, she developed status epilepticus. Follow-up MRI showed edematous changes in the cortex and deep white matter of the frontal, parietal, and occipital lobes, as well as in the basal ganglia and cerebellum, accompanied by hemorrhage and meningeal enhancement. Her serum test turned out to be positive for anti-aquaporin-4 antibody later. Finally, she was diagnosed with PRES accompanied by NMOSD. It is of note to consider NMOSD when patients with autoimmune diseases present with neurological symptoms such as optic neuritis or acute myelitis. Based on previous reports and temporal changes in her antibody titers, SS may have been involved in the onset of NMOSD in this case. When NMOSD complicates SS, it can be challenging to distinguish CNS lesions caused by SS from those caused by NMOSD. Although previous reports on the coexistence of NMOSD and PRES are limited, several factors have been proposed to explain the mechanism underlying this pathology.

## Introduction

Neuromyelitis optica spectrum disorder (NMOSD) is an autoimmune disease of the central nervous system (CNS) primarily affecting the optic nerve, spinal cord, and certain brain regions such as the area postrema [1]. The disease is characterized by the presence of anti-aquaporin-4 (AQP4) antibody. AQP4 is the principal water channel involved in fluid homeostasis in the CNS and is expressed in the area of abluminal surface of blood vessels and astrocytic foot processes. Anti-AQP4 antibody therefore targets astrocytes, leading to demyelination and severe neurological symptoms. This disease is similar to multiple sclerosis (MS) in that both are demyelinating disorders affecting the central nervous system. However, this differs from MS in that specific antibodies remain unknown in MS. NMOSD is increasingly recognized to coexist with other autoimmune diseases, such as systemic lupus erythematosus and Sjögren's syndrome (SS) [2], suggesting the involvement of shared pathogenic pathways. Notably, 6.5% of SS patients are reported to have NMOSD, while 7.0% of NMOSD patients have SS [2], indicating a relatively strong association compared to other autoimmune diseases.

Another unique aspect of NMOSD is its potential to be complicated by posterior reversible encephalopathy syndrome (PRES), a condition characterized by transient vasogenic edema commonly observed in the parieto-occipital regions. The pathology of PRES is thought to involve endothelial dysfunction. The coexistence of PRES and NMOSD is rare, with only 14 cases reported as of 2022 [3]. Proposed mechanisms underlying this comorbidity include blood-brain barrier dysfunction mediated by interleukin-6 (IL-6) and treatment-related effects such as plasma exchange or steroids [4-6].

We present here a case of NMOSD that developed in the clinical course of SS and autoimmune hepatitis (AIH), which was rapidly complicated by PRES. We also discuss the mechanisms underlying the coexistence of these diseases and the characteristics of imaging findings.

This article was previously presented as a meeting abstract at the 53rd annual meeting of the Japanese Society of Neuroradiology on February 9-10, 2024.

## Case presentation

The patient was a 28-year-old woman who had been receiving outpatient care at our hospital for SS and AIH for six years. Two weeks prior, she began experiencing nausea and singultus, which made it difficult for her to eat. Around the same time, she developed intermittent numbness from her neck to her upper arms. Due to an inability to eat and a weight loss of seven kilograms, she was admitted to the gastroenterology department of our hospital in February 2022 for further evaluation and treatment. On the third day of hospitalization, partial visual blurring in her left visual field appeared, which extended to the entire left visual field. On the fifth day, her vision in the left eye was impaired, with a diminished pupillary light reflex and an upper-half visual field defect. Intermittent sensory abnormalities were observed on both sides of the neck, upper arms, left side of the back, and left buttock.

**Figure 1:**
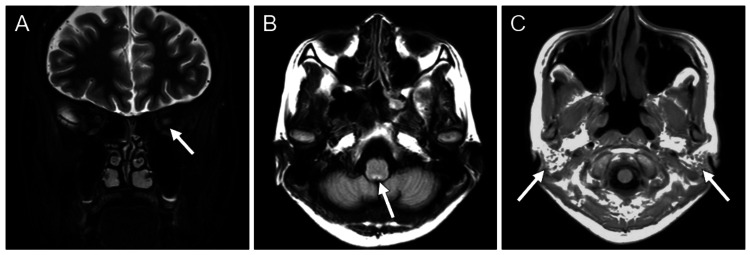
Brain magnetic resonance imaging on the fifth day of hospitalization. (A) Coronal short tau inversion recovery image shows enlargement and hyperintensity of the left optic nerve. (B) Axial fluid attenuated inversion recovery image reveals a hyperintense area in the area postrema. (C) Axial T1-weighted image shows atrophy, fatty infiltration, and punctate soft tissue signals in the parotid gland, reflecting Sjogren's syndrome.

Laboratory test results on the fifth day showed a white blood cell count of 5,290/μL and a C-reactive protein level of 0.01 mg/dL, both within normal inflammatory response ranges. Cerebrospinal fluid analysis revealed an elevated cell count of 78 cells/μL (100% monocytes) and a protein concentration of 59 mg/dL. Additionally, myelin basic protein was elevated at 525 pg/mL, and IL-6 was increased to 56.2 pg/mL. The IgG index was 0.65 and oligoclonal bands were negative (Table 1). Brain magnetic resonance imaging (MRI) on the fifth day showed enlargement and hyperintensity in the left optic nerve on short tau inversion recovery imaging (Figure 1A), and a hyperintense area in the area postrema on fluid attenuated inversion recovery (FLAIR) imaging (Figure 1B). Based on these imaging findings and the symptoms of myelitis, a tentative diagnosis of NMOSD was made. As differential diagnoses, MS and myelin oligodendrocyte glycoprotein antibody-associated diseases were considered; however, the lesion in the area postrema observed in this patient was characteristic of NMOSD. Even with this finding, central nervous system lesions associated with SS could not be entirely ruled out, though the combination with other lesions was more suggestive of NMOSD. Steroid therapy (prednisolone at 1,000 mg/day for three days, followed by maintenance at 20 mg/day) and plasmapheresis (twice weekly, for a total of seven sessions) were initiated the same day.

**Table 1:** Laboratory test results on the fifth day of hospitalization. * Anti-aquaporin-4 antibody is included in the diagnostic criteria for neuromyelitis optica spectrum disorder (NMOSD). SS: Sjögren's syndrome

Blood examination	Result	Reference Range
White blood cell count (/μL)	5,290	3,300-8,600
C-reactive protein (mg/dL)	0.01	≤0.14
Antinuclear antibody	1:160	≤1:40
Anti-SS-A antibody (U/mL)	10,900	≤9.9
Anti-SS-B antibody (U/mL)	868	≤9.9
Anti-aquaporin-4 antibody* (U/mL)	≥40	≤2.9
Cerebrospinal fluid analysis	Result	Reference Range
Cell count (/μL)	78	≤15
Protein concentration (mg/dL)	59	10-40
Myelin basic protein (pg/mL)	525	≤102
Interleukin-6 (pg/mL)	56.2	≤4
IgG index	0.65	<0.73
Oligoclonal bands	Negative	Negative

**Figure 2:**
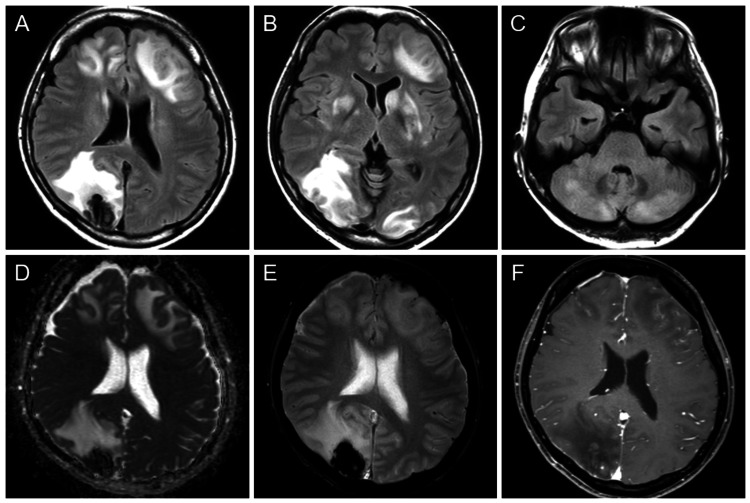
Brain magnetic resonance imaging performed after the seizure on the ninth day of hospitalization. (A-C) Fluid attenuated inversion recovery images show hyperintensity and swelling in multiple areas, including the cortex and deep white matter of the frontal, parietal, and occipital lobes, as well as the basal ganglia and cerebellum. (D-F) The lower-row figures represent apparent diffusion coefficient (ADC) map (D), T2*-weighted image (E), and contrast-enhanced fat-suppressed T1-weighted image (F), all corresponding to the same slice level as shown in Panel A. The lesions are accompanied by hemorrhage and meningeal enhancement. The non-hemorrhagic areas show increased ADC values.

On the sixth day of hospitalization, the patient developed status epilepticus. She developed impaired communication accompanied by right conjugate gaze deviation and seizures in the left upper and lower extremities. Electroencephalography revealed the disappearance of the posterior dominant rhythm and irregular generalized slow waves predominantly in the right frontal region. During the seizures, her blood pressure transiently increased from 108/82 mmHg to 155/90 mmHg. A postictal brain MRI showed hyperintensity and swelling in multiple areas, including the cortex and deep white matter of the frontal, parietal, and occipital lobes, as well as the basal ganglia and cerebellum on FLAIR imaging (Figure 2A-2C). The lesions demonstrated high apparent diffusion coefficient (ADC) values and were accompanied by hemorrhage and meningeal enhancement (Figure 2D-2F). Based on these MRI findings, her background of autoimmune disease, and transient blood pressure elevation, we suspected that this case was NMOSD complicated by PRES. The differential diagnoses included postictal encephalopathy and acute disseminated encephalomyelitis (ADEM). Postictal encephalopathy was ruled out due to discrepancies in imaging findings, and ADEM was considered unlikely given the atypical age of onset and the lack of preceding infectious episodes. Anticonvulsants (fosphenytoin, levetiracetam, and lacosamide) were administered for the following four days to control the seizures as follows: from the sixth to the ninth days, adjustments were made to the doses of fosphenytoin (750 mg → 300 mg → 337.5 mg), levetiracetam (1,000 mg → 2,000 mg), and lacosamide (400 mg → 200 mg). Levetiracetam was increased on the ninth day, and lacosamide was initiated on the eighth day and reduced on the ninth day. For the NMOSD, steroid therapy and plasmapheresis were continued. Since the blood pressure elevation was transient, no specific intervention was required.

**Figure 3:**
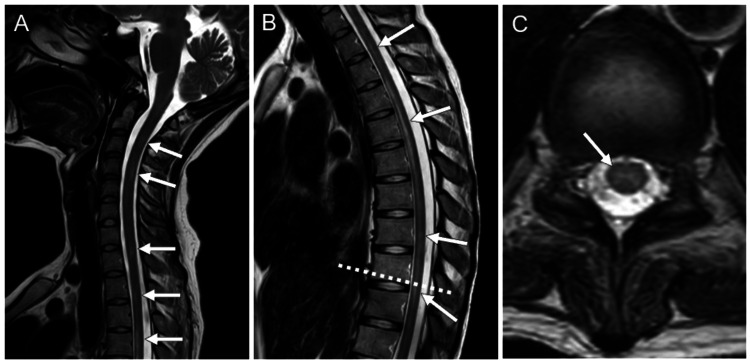
Cervical and thoracic spine magnetic resonance imaging. (A) Sagittal T2-weighted image of the cervical spine reveals hyperintense lesions in the spinal cord. (B) Sagittal T2-weighted image of the thoracic spine demonstrates hyperintense lesions in the spinal cord. The lesions extend over more than three vertebral segments, satisfying the criteria of neuromyelitis optica spectrum disorder (NMOSD). (C) Axial T2-weighted image at the level of the dotted line in Panel B shows a hyperintense lesion predominantly located in the central gray matter.

The results of various antibody tests on the fifth day were obtained several days later, revealing a positive anti-AQP4 antibody result (≥40 U/mL). Antinuclear antibody was also elevated at a titer of 1:160, with anti-SS-A antibody at 10,900 U/mL and anti-SS-B antibody at 868 U/mL (Table 1). Cervical spine MRI performed on the 10th day of hospitalization and thoracic spine MRI on the 11th day revealed hyperintense lesions in the spinal cord from the C2 to Th10 vertebral levels on T2-weighted imaging, including lesions extending over more than three vertebral segments (Figure 3). Given the positive anti-AQP4 antibody results, along with the presence of optic neuritis, a lesion in the area postrema, and acute myelitis, a diagnosis of NMOSD was made. 

**Figure 4:**
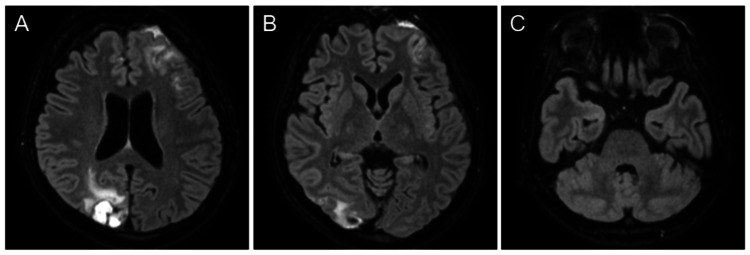
Brain magnetic resonance imaging on the 29th day of hospitalization. (A-C) Fat-suppressed three-dimensional fluid attenuated inversion recovery images reveal that the lesions exhibit a tendency to resolve.

Follow-up brain MRI on the 29th day of hospitalization showed resolution of the lesions (Figure 4), consistent with the natural course of PRES. The patient's visual impairment and sensory abnormalities in the limbs, symptoms attributed to NMOSD, gradually improved. Although she experienced muscle weakness and sensory disturbances in the lower extremities, as well as bladder and bowel dysfunction during hospitalization, these symptoms had improved by the time of discharge about one month later.

In summary, this case was finally diagnosed as NMOSD complicated by PRES. The positive anti-AQP4 antibody, optic neuritis, acute myelitis, and the lesion in the area postrema met the diagnostic criteria for NMOSD. Additionally, the patient developed status epilepticus, and PRES was suspected based on the clinical course and MRI findings. The follow-up MRI showed improvement consistent with the course of PRES. 

## Discussion

NMOSD was affirmed as a concept encompassing a group of diseases related to anti-AQP4 antibody by the International Panel for NMO Diagnosis (IPND) in 2015 [1]. Traditional neuromyelitis optica (NMO) is an inflammatory demyelinating disorder characterized by optic neuritis and myelitis. Previously considered as a subtype of MS, NMO was distinguished as a separate disease with the discovery of anti-AQP4 antibody specific to NMO. In 2007, the term NMOSD was introduced to include anti-AQP4 antibody-positive patients with limited or inaugural forms of NMO (e.g., first-attack longitudinally extensive transverse myelitis (LETM) or recurrent or bilateral optic neuritis) who were at high risk for future attacks [7]. In 2015, NMO and NMOSD were unified under the name of NMOSD by the IPND above. The characteristic features of NMOSD include MRI findings related to optic nerve, spinal cord, area postrema, other brainstem, diencephalic, or cerebral presentations [1].

The primary pathology of NMOSD involves astrocyte damage caused by anti-AQP4 antibody, and an association between NMOSD and other autoimmune diseases has been recognized [2]. It has been reported that up to 20-30% of patients with NMO are associated with autoimmune diseases [8]. Although reports on NMOSD remain limited, a study revealed that 45 out of 180 patients with NMOSD had comorbid autoimmune diseases [9]. One proposed mechanism for this association suggests that these autoimmune diseases induce the production of inflammatory cytokines, which in turn activate AQP4-specific T cells, leading to the onset of NMOSD [10].

Here, we report a patient with a history of SS and AIH who developed NMOSD. A systematic review and meta-analysis found that 6.5% of SS patients had NMOSD, while 7.0% of NMOSD patients had SS [2]. In contrast, comorbidity of AIH and NMOSD is limited to case reports, suggesting that the association of NMOSD with AIH is less common than with SS. In this case, antibody titers for antinuclear, anti-SS-A, and anti-SS-B antibodies were regularly monitored as indicators of SS and AIH. Typically, antinuclear antibody is positive in both AIH and SS, while anti-SS-A and anti-SS-B antibodies are positive in SS. Before the onset of NMOSD, the patient’s antibody titers were 1:160 for antinuclear antibody, 131 U/mL for anti-SS-A antibody, and 96.5 U/mL for anti-SS-B antibody. However, at the time of NMOSD onset, the antinuclear antibody titer remained at 1:160, but anti-SS-A and anti-SS-B antibodies showed a marked increase to 10,900 U/mL and 868 U/mL, respectively. These findings suggest that SS may have played a more significant role in the onset of NMOSD in this context, compared to AIH.

The patient was positive for anti-AQP4 antibody and also exhibited optic neuritis, acute myelitis, and area postrema syndrome, leading to the diagnosis of NMOSD. When NMOSD coexists with SS, as observed in the patient, it becomes challenging to distinguish CNS lesions caused by SS from those caused by NMOSD. CNS involvement in SS often mimics MS, but cases presenting with only optic neuritis [11], LETM [12], or lesion of medulla oblongata [13] have also been reported. These are the characteristic areas frequently involved in NMOSD [1], thus differentiation from NMOSD on imaging alone can be difficult. While elevated antibody titers specific to SS and NMOSD can confirm the presence of each disease, it remains difficult to determine whether those CNS lesions were attributed to SS or NMOSD. As for the prognosis, Akaishi et al. reported on the impact of SS comorbidity in anti-AQP4 antibody-positive NMOSD patients [14], noting that the relapse frequency in patients with anti-AQP4 antibody-positive NMOSD increased with comorbid SS. Thus, careful monitoring for recurrence will be necessary in our case, though no relapse has been observed two years and six months after the onset.

PRES is characterized by reversible vasogenic edema predominantly in the parieto-occipital regions, leading to acute or subacute neurological symptoms such as headaches, visual disturbances, and seizures. Risk factors for PRES include acute hypertension, autoimmune diseases, renal failure, immunosuppressive agents, and pregnancy. Brain MRI typically shows hyperintense areas in the cortex and subcortical regions of the bilateral parieto-occipital lobes on T2-weighted imaging and FLAIR imaging, with increased ADC values reflecting vasogenic edema, sometimes accompanied by hemorrhage and meningeal enhancement. Classically, two main theories for the pathogenesis of PRES have been proposed: severe hypertension disrupting brain autoregulation, resulting in endothelial edema or injury [6]; systemic toxicity from inflammatory cytokines leading to endothelial dysfunction [15].

This case was diagnosed as NMOSD complicated by PRES. The co-occurrence of NMOSD and PRES has been reported in 14 cases as of 2022 [3], and no further cases were identified through 2024 in a PubMed search. Several factors are thought to contribute to this comorbidity [4-6,16,17]: (1) anti-AQP4 antibody induces IL-6 production by AQP4-positive astrocytes, and IL-6 signaling to vascular endothelial cells decreases the blood-brain barrier function [4]; (2) water flux impairment due to AQP4 autoimmunity may predispose to PRES in patients with NMOSD who experience blood pressure fluctuations, or who are treated with therapies that can cause rapid fluid shifts such as intravenous immunoglobulin and plasma exchange [5]; (3) steroids, frequently used for the treatment of NMOSD, are risk factors for PRES [6]; (4) glucose-regulated protein 78 (GRP78) autoantibody associates with blood-brain barrier disruption in NMO [17]. In the present patient, IL-6 levels in the cerebrospinal fluid were elevated to 56.2 pg/mL, and the patient developed PRES the day after the initiation of steroid therapy and plasmapheresis, with concurrent blood pressure elevation, thus a combination of mechanisms (1) to (3) may have contributed. Notably, the close temporal proximity between the initiation of treatments for NMOSD and the onset of PRES suggests that these treatments may have significantly related to the development of PRES. However, it remains unclear what factors contribute and to what extent, especially when considering the rarity of this comorbidity. In this case, PRES symptoms were tolerated with transient elevation of blood pressure and response to anticonvulsants, and steroid therapy and plasmapheresis were continued with careful monitoring; subsequent symptoms were limited, and no more special intervention was required. GRP78 antibody was not measured in this patient, so the involvement of mechanism (4) remains unclear.

In this case, hyperintense signals and swelling were observed in the frontal, parietal, and occipital lobes, as well as the basal ganglia and cerebellum on FLAIR imaging at the time of PRES onset. A report summarizing 14 cases of NMOSD complicated by PRES found that the bilateral temporal, occipital, and parietal lobes were the most common lesion sites [3]. In addition to these common areas, lesions were found in the frontal lobes, basal ganglia, and cerebellum in this patient. These regions are also recognized as less frequent but potential locations for PRES in general, thus we cannot insist that these are key image findings for PRES with NMOSD.

## Conclusions

We reported a case of NMOSD that developed in the context of SS and AIH, thereafter complicated by PRES. NMOSD can occur in association with other autoimmune diseases, and it is important to recognize NMOSD based on imaging findings, though it may be difficult to differentiate from CNS lesions of SS. The mechanisms by which NMOSD coexists with PRES are varied. Treatments for NMOSD such as steroids and plasma exchange may possibly cause the development of PRES, thus careful monitoring of the treatment is essential.
